# Natural history and outcome of 200 outpatients with classical trigeminal neuralgia treated with carbamazepine or oxcarbazepine in a tertiary centre for neuropathic pain

**DOI:** 10.1186/1129-2377-15-34

**Published:** 2014-06-09

**Authors:** Giulia Di Stefano, Silvia La Cesa, Andrea Truini, Giorgio Cruccu

**Affiliations:** 1 Neuropathic Pain Unit, Department of Neurology and Psychiatry, Sapienza University, viale Università 30, 00185 Roma, Italy

**Keywords:** Classic trigeminal neuralgia, Natural history, Carbamazepine, Oxcarbazepine, Tolerability

## Abstract

**Background:**

The guidelines on trigeminal neuralgia management that have been agreed and jointly published by the American Academy of Neurology and the European Federation of Neurological Societies recommend carbamazepine (CBZ) and oxcarbazepine (OXC) as the first-choice medical treatments in patients with trigeminal neuralgia (TN). The aim of this retrospective study was to analyze the natural history of classical trigeminal neuralgia in a large cohort of patients, focusing on drug responsiveness, side effects related to CBZ and OXC, and changes in pain characteristics during the course of disease.

**Findings:**

We selected the last 100 consecutive patients with typical TN who began treatment with CBZ and the last 100 with OXC. All had MRI scans and a complete neurophysiological study of trigeminal reflexes. Among them, 22 were excluded on the basis of neuroradiological or neurophysiological investigations, to avoid the inclusion of patients with possible secondary TN. The initial number of responders was 98% with CBZ with a median dose of 600 mg (range 200–1200), and of 94% with OXC, with a median dose of 1200 mg (range 600–1800). In a mean period of 8.6 months, 27% of responders to CBZ incurred in undesired effects to a level that caused interruption of treatment or a dosage reduction to an unsatisfactory level. In a mean period of 13 months, the same occurred to 18% of responders to OXC. Among patients who had a good initial response, only 3 patients with CBZ and 2 with OXC developed late resistance. During the course of disease, paroxysms worsened in intensity in 3% of patients, and paroxysms duration increased in 2%. We did not observe the onset of a clinically manifest sensory deficit at any time in any patient.

**Conclusions:**

Unlike common notion, in our large patient sample the worsening of pain with time and the development of late resistance only occurred in a very small minority of patients. CBZ and OXC were confirmed to be efficacious in a large majority of patients, but the side effects caused withdrawal from treatment in an important percentage of patients. These results suggest the opportunity to develop a better tolerated drug.

## Introduction

Trigeminal Neuralgia (TN) is a clinical condition characterized by a sudden, usually unilateral, brief, stabbing, recurrent pain with a distribution consistent with one or more divisions of the fifth cranial nerve [1]. The paroxysmal attacks are stereotyped in the individual patient, last from a fraction of a second to 2 minutes [2] and may be evoked by stimulating cutaneous or mucous trigeminal territories, the so-called trigger zones. The pain-free intervals may range from days to years [3]. TN may be distinguished in classical, namely without a cause other than a neurovascular compression, or secondary to a demonstrable lesion, including benign tumors of the cerebellopontine angle or multiple sclerosis [4-6]. According to the symptom constellation, TN is categorized into typical and atypical form, the latter characterized by a constant and non-lancinating background pain and, sometimes, sensory disturbances in the affected division [7,8]. The annual age-adjusted incidence is 5.9% for women and 3.4% for men [9].

According to the American Academy of Neurology (AAN), the European Federation of Neurological Societies (EFNS) and also other recent guidelines, carbamazepine (CBZ) and oxcarbazepine (OXC) are the first-line medical treatments for pain control in patients with TN [10-12]. They have the same mechanism of action, namely the blockade of voltage-gated sodium channels in a frequency-dependent manner. OXC may be preferred because of the minor risk for drug interactions and its better tolerability in comparison with CBZ [11]. The AAN-EFNS guidelines recommended that patients unresponsive or that cannot reach the therapeutic dosage of the drug because of adverse events should be made aware of the availability of surgery. Surgical procedures include Gasserian ganglion percutaneous techniques, microvascular decompression in the posterior fossa, and gamma knife radiosurgery. These procedures are extremely efficacious with relatively few complications. Microvascular decompression may be considered over other surgical techniques to provide the longest duration of pain freedom [10]. According to the available evidence no oral treatment is better than CBZ or OXC, but in case of refractory trigeminal neuralgia, among the non-surgical option, lamotrigine and botulinum toxin injections should be considered [13].

Up to now, only few studies have focused on the development of the clinical picture and the drug efficacy and tolerability in time. The aim of this retrospective study was to analyze the natural history of TN in a large cohort of patients, by focusing on the drug responsiveness, the side effects related to the pharmacological treatment, the changes in pain characteristics along with the duration of the disease, such as duration and intensity of paroxysms, and the possible onset of sensory disturbances.

## Methods

The staff nurse retrospectively selected the clinical notes of outpatients with a diagnosis of classical TN who had attended our centre for neuropathic pain from January 2000 to June 2013. One of us analyzed the clinical notes and selected the last consecutive 100 patients who began treatment with CBZ and the last consecutive 100 who did it with OXC. All patients included in the analysis suffered from paroxysmal attacks of pain lasting from a fraction of a second to 2 minutes, affecting one or more divisions of the trigeminal nerve. Pain was described as intense, sharp, superficial or stabbing; paroxysms could be both spontaneous and precipitated from trigger areas or by trigger factors. The attacks were stereotyped in the individual patient. The clinical examination did not show any clinical neurological deficit [2]. All patients had undergone MRI scans of the brain and trigeminal reflex testing [4,6,10] in order to identify with certainty even patients with typical presentation but a possibly secondary origin, including idiopathic sensory trigeminal neuropathy and nerve trauma [14].

Patients were seen at least monthly until the target dosage and/or a significant pain reduction was reached. Then, follow-up visits were scheduled every six months, unless side effects occurred.

Two staff members were involved in the clinical examination and two in the neurophysiological testing. The diagnosis of classical trigeminal neuralgia was confirmed by at least two clinicians.

We focused our attention on the average onset age of TN, the number of responders to CBZ or OXC, the possible CBZ/OXC lost efficacy, the side effects that caused interruption of treatment or a dosage reduction to an unsatisfactory level and the latency for the side effect onset. Patients were considered as responders on the bases of their global satisfaction and the willingness to continue the drug.

We also analyzed the possible change in pain characteristics during the course of disease, including paroxysms duration and intensity. By definition, classical TN is a pain syndrome that arises without a clinically manifest sensory deficit: anyway, we wanted to test the likelihood of the onset of sensory disturbances during the disease course.

## Findings

We considered an otherwise homogeneous group of 200 patients (68 M, 132 F, mean age 67.54 ± 12.11), with a mean follow-up period of 7.31 years. Among them, 22 patients with typical symptom constellation and a diagnosis of classical TN were excluded from the study because two of us considered the results of neurophysiological or neuroimaging investigations insufficient to exclude a secondary form with absolute certainty. The other 178 patients had a classical TN, without any evidence of a cause other than a neurovascular conflict at dedicated MRI scans. Ninety-five out of 178 patients were treated with CBZ and the remaining 83 with OXC. The average onset age of symptoms was 60 ± 11.6 years (range 35-80).

**Figure 1:**
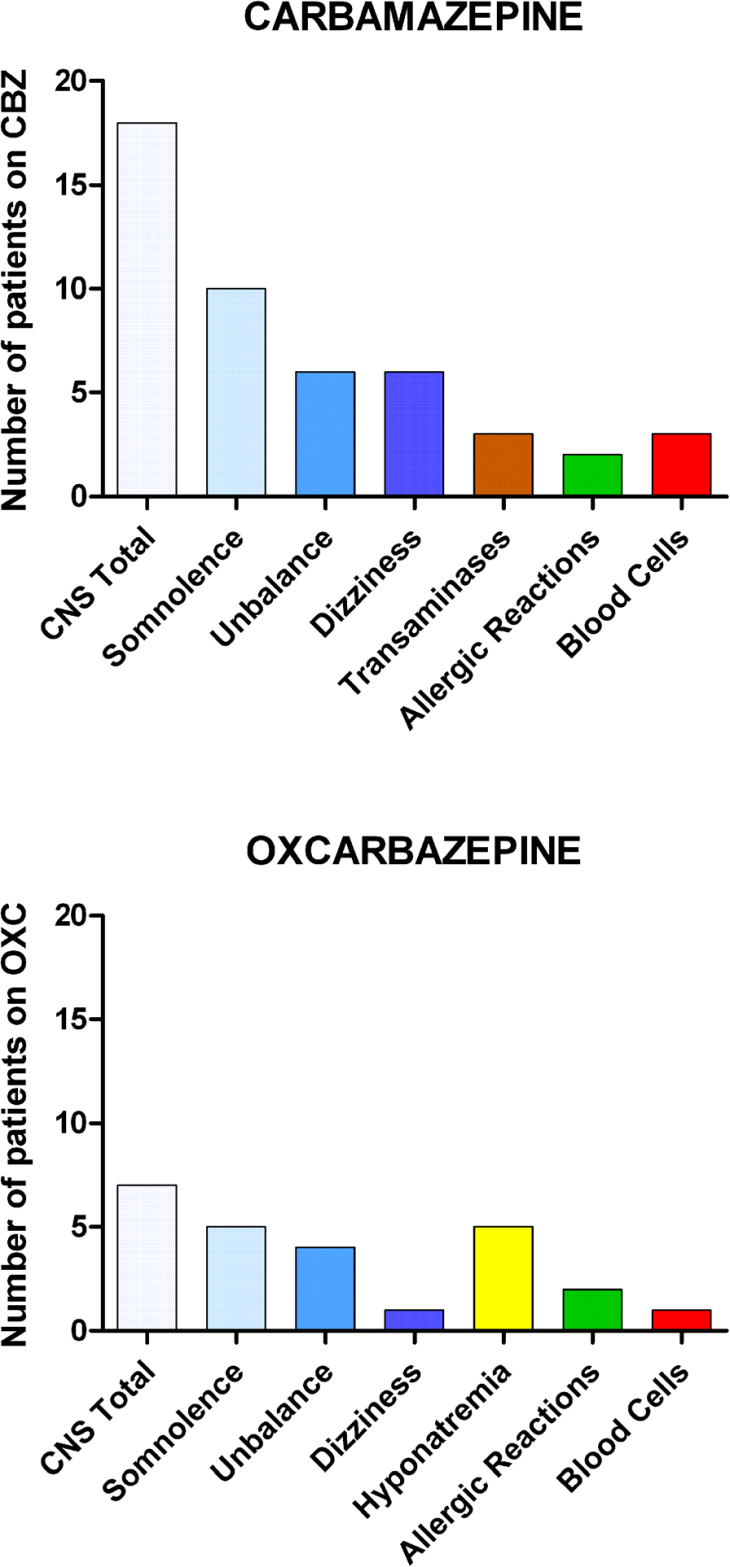
**Tolerability of Carbamazepine (CBZ) and Oxcarbazepine (OXC).** Y-axis: number of patients that discontinued first treatment because of adverse events. X-axis: causes of discontinuation. Note that CNS disturbances affected far more frequently the patients on CBZ, whereas hyponatremia only affected patients on OXC. The sum of patients reporting somnolence, postural unbalance, and dizziness is higher than the total CNS disturbances because many patients complained of more than one CNS disturbance.

The initial number of responders was 98% with CBZ at a median dosage of 600 mg (range 200–1200 mg), and of 94% with OXC at a median dosage of 1200 mg (range 600–1800 mg). Among responders to CBZ, 27% of patients incurred in adverse events that directly caused interruption of treatment by the physician or a dosage reduction to a level that was insufficient to control pain, thus causing discontinuation, after a mean period of 8.6 months. In a mean period of 13 months, physician- or patient-decided discontinuations occurred in 18% of patients initially responders to OXC.The causes of these discontinuations are plotted in Figure 1. The CNS disturbances were about triple in patients on CBZ than those on OXC. In detail, the CNS disturbances included somnolence (10 patients treated with CBZ and 5 with OXC), postural unbalance (6 with CBZ and 4 with OXC) and dizziness (6 with CBZ and 1 with OXC). Among patients under CBZ, three had an increase of transaminases, one anemia, one leucopenia, and one thrombocytopenia. Among those under OXC, 5 had hyponatremia, one patient had thrombocytopenia. Allergic reactions (cutaneous rash) affected two patients on CBZ and two on OXC.

The onset of side effects on the CNS occurred with a mean dosage of 600 mg for CBZ and 1200 mg for OXC. With CBZ, anaemia, leucopenia, and thrombocytopenia occurred within the first three weeks of treatment, with dosages of 600 mg, 600 mg, and 1000 mg, respectively. With OXC, thrombocytopenia occurred within the first two weeks of treatment, with a dosage of 1200 mg. Hyponatremia was observed within the first month, with dosages of 900 mg in one patient, 1200 mg in three patients and 1800 mg in the fifth patient. Allergic reactions occurred at the beginning of treatment, i.e. within the first two days. In this case the treatment was interrupted immediately. Among patients that had to interrupt CBZ because of adverse events, 16 were switched to OXC, and 5 to gabapentin. The new treatment was effective in 12 patients (10 with OXC and 2 with gabapentin), whereas in 7 patients (6 with OXC and 1 with gabapentin) the new treatment was unsuccessful. The remaining two patients were lost to follow up. Among patients that had to interrupt OXC because of adverse events, 3 were switched to CBZ, and 2 to gabapentin. The new treatment was effective in 4 patients (3 with CBZ and 1 with gabapentin), whereas in the remaining patient the new treatment was unsuccessful. Eight patients were lost to follow up. Eventually, 13 patients out of 178 were referred for surgery (5 patients treated with OXC and 8 with CBZ). Among patients who had a good initial response, three patients with CBZ and two with OXC developed late resistance after 24–76 months. The intensity of paroxysms worsened in six patients and their duration in four. Such a worsening occurred in a mean time of 55 months. Constant pain developed in 5 patients, after an average duration of disease of 7 years. We did not observe the development of sensory disturbances with time in any patient suffering from classical TN.

## Discussion

In this retrospective study in a large cohort of patients, we investigated the natural history of classical TN, focusing our attention on the efficacy of CBZ or OXC, the possible onset of a late resistance and the side effects that eventually caused either interruption of treatment or a dosage reduction to an unsatisfactory level. The possible modifications in pain characteristics during the course of disease, including paroxysms duration and intensity, were also examined.

We found that the worsening of pain with time and the development of late resistance only occurred in a very small minority of patients. CBZ and OXC were confirmed to be efficacious in a large majority of patients, but the side effects caused the withdrawal from treatment in an important percentage of patients.

Demographics matched those observed in previous studies [9], with a higher frequency in women (65.17%) and an average onset age at 60 years.

We found changes in pain characteristics only in an extremely small sub-set of patients, compared to the total number. Such changes included the increase of both paroxysms duration and intensity. Unlike other reports [15], no sensory deficit was observed since the beginning of the disease.

It is generally agreed that the first line therapy of TN is pharmacological and based on the use of sodium channels blockers, CBZ and OXC. Four placebo-controlled trials demonstrated the efficacy of CBZ [16-19] with a number needed to treat to obtain important pain relief of 1.7-1.8 [20]. This efficacy is however compromised by the tolerability, with a numbers needed to harm of 3.4 for minor and of 24 for severe adverse events [21,22]. OXC has a comparable efficacy to that of CBZ but a greater tolerability and a lower potential for drug interaction [23-25].

This study confirmed that CBZ and OXC are efficacious in a great majority of patients and that OXC is more tolerated in comparison with CBZ. If compared with other reports, the percentage of non responders was somewhat lower in our sample. Because CBZ and OXC are extremely efficacious in increasing the refractory period of action potentials, they are bound to be most active on the high-frequency discharges that characterize the paroxysms of trigeminal neuralgia. Naturally, if the patient selection is not very strict, and concedes the recruitment of a few patients that also have some ongoing pain, then the efficacy of CBZ/OXC may drop. Indeed, the diagnostic accuracy has always been a problem in studies in trigeminal neuralgia.Adverse events may cause withdrawal from treatment. This occurred in a significant amount of patients, 27% of those with CBZ and 18% of those with OXC, who were initially responders. The most frequent adverse effects involved the central nervous system, and included somnolence, dizziness and postural unbalance. CBZ had a higher percentage of discontinuations for all kinds of side effect, except for sodium depletion, which only occurred with OXC (Figure 1).

Although in our centre we are well aware of the great efficacy of surgical interventions for trigeminal neuralgia [10,11] and we always offer this chance to patients resistant to CBZ/OXC, only 7% of this large cohort of patients was eventually sent for surgery, a proportion decidedly low if compared to those reported by neurosurgical centres. To explain the low proportion of patients sent for surgery, we may think of this main explanation: the local population is not so keen on undergoing surgery unless they really cannot manage with medical treatment and the number of patients resistant to CBZ/OXC was very low.

In conclusion, the failure of the treatment with CBZ/OXC, most of the times, is not due to the inefficacy of the drug, but rather to undesired effects to a level that causes interruption of treatment or a dosage reduction to an insufficient level. These results suggest the opportunity to develop a better tolerated drug.

## Abbreviations

TN: Trigeminal nevralgia; CBZ: Carbamazepine; OXC: Oxcarbazepine; AAN: American Academy of Neurology; EFNS: European Federation of Neurological Societies.

## Competing interests

None of the authors have received financial support for the generation of this article. The authors have no personal or institutional interest in any of the drugs mentioned in this article.

## Authors’ contributions

Conception, design of the work, data acquisition and analysis, data interpretation and draft of the article were done by all authors. All the authors have read and approved the final manuscript.
